# FluoNeRF: Fluorescent Novel-View Synthesis Under Novel Light Source Colors and Spectra †

**DOI:** 10.3390/jimaging12010016

**Published:** 2025-12-29

**Authors:** Lin Shi, Kengo Matsufuji, Michitaka Yoshida, Ryo Kawahara, Takahiro Okabe

**Affiliations:** 1 Department of Artificial Intelligence, Kyushu Institute of Technology, Fukuoka 820-8502, Japan; matsufuji.kengo601@mail.kyutech.jp; 2 Research Center for Neuromorphic AI Hardware, Kyushu Institute of Technology, Fukuoka 808-0196, Japan; 3 Department of Computer Science, Okayama University, Okayama 700-8530, Japan; michitaka-yoshida@okayama-u.ac.jp; 4 Graduate School of Informatics, Kyoto University, Kyoto 606-8501, Japan; ryo@vision.ist.i.kyoto-u.ac.jp

**Keywords:** novel-view synthesis, neural radiance fields, relighting, superposition principle, fluorescence, Stokes shift

## Abstract

Synthesizing photo-realistic images of a scene from arbitrary viewpoints and under arbitrary lighting environments is one of the important research topics in computer vision and graphics. In this paper, we propose a method for synthesizing photo-realistic images of a scene with fluorescent objects from novel viewpoints and under novel lighting colors and spectra. In general, fluorescent materials absorb light with certain wavelengths and then emit light with longer wavelengths than the absorbed ones, in contrast to reflective materials, which preserve wavelengths of light. Therefore, we cannot reproduce the colors of fluorescent objects under arbitrary lighting colors by combining conventional view synthesis techniques with the white balance adjustment of the RGB channels. Accordingly, we extend the novel-view synthesis based on the neural radiance fields by incorporating the superposition principle of light; our proposed method captures a sparse set of images of a scene from varying viewpoints and under varying lighting colors or spectra with active lighting systems such as a color display or a multi-spectral light stage and then synthesizes photo-realistic images of the scene without explicitly modeling its geometric and photometric models. We conducted a number of experiments using real images captured with an LCD and confirmed that our method works better than the existing methods. Moreover, we showed that the extension of our method using more than three primary colors with a light stage enables us to reproduce the colors of fluorescent objects under common light sources.

## 1. Introduction

Fluorescence is a very common phenomenon observed both in natural objects, such as minerals and plants, and in man-made objects, such as papers and clothes [1,2]. In general, fluorescent materials absorb light with certain wavelengths and then emit light with longer wavelengths than the absorbed ones. This property, called *Stokes shift,* is in contrast to that of reflective materials, which reflect light with the same wavelengths as those of the incident light.

Novel-view synthesis, i.e., synthesizing photo-realistic images of a scene from arbitrary viewpoints, is useful for XR (extended reality/cross reality) and is one of the important research topics in computer vision and graphics. Recently, novel-view synthesis based on NeRF (neural radiance fields) [3] has achieved great success, and the extension of the original NeRF is actively studied. In this paper, we propose a method for synthesizing photo-realistic images of a scene with *fluorescent objects* from novel viewpoints and under *novel lighting colors and spectra*.

**Figure 1:**
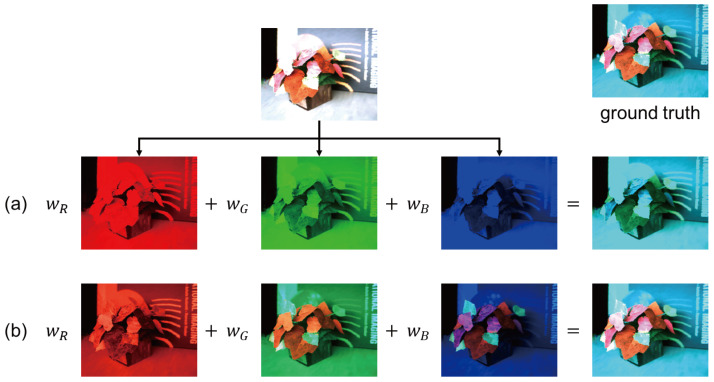
Key observation: (**a**) the white balance adjustment, i.e., the linear combination of the R, G, and B channels of an image taken under a white light source, cannot reproduce the colors of fluorescent objects under a novel light source color, but (**b**) the linear combination of three images taken under R, G, and B light sources works well.

For reflective materials, we can approximately reproduce the colors of the objects under arbitrary lighting conditions by combining conventional view synthesis techniques with the white balance adjustment of the RGB channels. This is because the image of the B channel taken under a white light source, for example, is almost the same as the image taken under a blue light source when we assume a narrow-band camera. However, it is not the case for fluorescent materials; we observe various colors other than blue due to the Stokes shift of fluorescence in the image taken under a blue light source. Figure 1a demonstrates that the white balance adjustment, i.e., the linear combination of the R, G, and B channels of an image taken under a white light source, cannot reproduce the colors of fluorescent objects under a novel light source color (see Appendix A and Appendix B for the theory behind such observation).

Accordingly, we extend the NeRF-based novel-view synthesis by incorporating the superposition principle of light. It says that an image taken under multiple light sources (a novel light source color or spectrum in our case) is represented as a linear combination of the images, each of which is taken under one of the light sources (three or more than three primary light source colors in our case). Figure 1b demonstrates that the linear combination of three images taken under R, G, and B light sources works well. Specifically, our proposed method captures a sparse set of images of a scene with fluorescent objects from varying viewpoints and under varying light source colors by using active lighting systems such as a color display with three primary colors (the primary light sources are polychromatic in general) and then synthesizes photo-realistic images of the scene without explicitly modeling the geometric and photometric models of the scene. Moreover, in order to improve the resolution and range of light source spectra represented by the primary colors, we extend our method by leveraging more than three primary light source colors (we approximate the continuous spectrum of a target light source by the linear combination of the spectra of narrow-band LEDs).

We conduct a number of experiments using real images and confirm the effectiveness of our proposed method. Specifically, we show that our method works better than the combination of the original NeRF with the white balance adjustment and the state-of-the-art methods. In addition, we show the validity of our proposed network that shares the volume densities among three primary light source colors. Moreover, we show that our method, based on the superposition principle, can accurately reproduce the colors of reflective objects under arbitrary lighting colors as a byproduct. Furthermore, we experimentally show with a multi-spectral light stage that the extension using more than three primary light source colors enables us to reproduce the colors of fluorescent objects under common light sources, such as daylight, which cannot be fully represented by a linear combination of three primaries.

The main contributions of this study include the following points:

- We address a novel problem of fluorescent novel-view synthesis under novel lighting colors and spectra.
- We propose a novel NeRF-based method by incorporating the superposition principle of light without explicitly modeling the geometric and photometric models of a scene of interest.
- Through a number of experiments with a color display, we confirm the effectiveness of our proposed method with shared volume densities.
- We show that our method performs better than the methods using the white balance adjustment, not only for fluorescent objects but also for reflective objects.
- In order to improve the resolution and range of light source spectra, we extend our method by leveraging more than three primary light source colors.
- Through a number of experiments with a multi-spectral light stage, we show the effectiveness of the extension using more than three primary colors.

## 2. Related Work

The original NeRF [3] implicitly models the radiance field of a scene by using an MLP (multilayer perceptron). Specifically, the MLP is a function that regresses a single volume density and view-dependent radiance values (RGB color) from the 3D position and 2D direction of a point in the scene. Since it achieves great success for novel-view synthesis, its extension is actively studied.

The original NeRF assumes a static scene consisting of reflective materials under a fixed lighting environment, and therefore, the extension includes the methods for dynamic scenes [4,5,6,7,8,9,10], non-reflective materials [11,12,13,14,15], and varying lighting environments [16,17,18,19,20,21,22,23,24,25,26,27,28]. In addition, the methods for improving the image quality and computational cost of novel-view synthesis [29,30,31,32,33,34,35,36] are proposed. Since the space is limited, we focus on the extension to non-reflective materials and varying lighting environments here.

### 2.1. Non-Reflective Materials

The extension to non-reflective materials includes transparent materials such as glass [11,12] and scattering media such as underwater scenes [13,14,15]. In contrast to the original NeRF, which mainly handles reflection and occlusion, those extensions need to consider additional physical phenomena. Specifically, the former takes both reflection and refraction into consideration, and the latter deals with scattering and absorption as well.

In this paper, we consider the extension to fluorescent materials by taking the physical phenomena inherent to fluorescent materials, i.e., absorption and emission, into consideration. There are a number of existing methods for fluorescent separation [2,37,38], spectral recovery [39], and shape recovery [40], but novel fluorescent-view synthesis under novel lighting colors and spectra is an open problem.

### 2.2. Varying Lighting Environment

One approach to synthesizing images under novel lighting environments, i.e., relighting is to recover the shape, BRDFs, and illumination of a scene of interest. The existing methods [16,19,20,22,24,25,27,28] decompose the image collection of a scene into the 3D shape, spatially varying BRDFs, and omnidirectional illumination environments, and then achieve novel-view synthesis under novel lighting environments. Unfortunately, however, we cannot apply those methods to scenes with fluorescent objects, because fluorescent materials depend on both incident and outgoing wavelengths and are described by not BRDFs but bispectral BRDFs [41].

The other approach is to learn the light transport of a scene from the image collection captured by using a light stage [23,42,43]. Such an approach has the advantage that it can directly capture the effects of global illumination. Unfortunately, however, the existing methods use multi-directional light sources with a fixed light source color (white) and then cannot capture the bispectral BRDFs of fluorescent materials. To cope with the above problem, our proposed method uses active light sources such as a color display or a multi-spectral light stage in addition to a camera and changes the light source colors or spectra illuminating a scene.

## 3. Proposed Method

In this section, we introduce the superposition principle on which our proposed method is based and then explain the pipeline of our method and our proposed network.

### 3.1. Superposition Principle

The superposition principle of images is often used in the computer vision community [44]. The superposition principle says that an image taken under multiple light sources is represented by the linear combination of the images, each of which is taken under one of the light sources. In our case, the image i of a scene under a novel light source color, i.e., a mixture of the three primary colors (R, G, and B) of light sources, is represented by the linear combination of the images $i_{R}$, $i_{G}$, and $i_{B}$ of the scene under the three primary light source colors as

(1) $$i=w_{R}i_{R}+w_{G}i_{G}+w_{B}i_{B}.$$

Here, $w_{R}$, $w_{G}$, and $w_{B}$ are the coefficients of the linear combination and depends on the color of the novel light source.

The validity of the superposition principle in fluorescence image formation relies on several key assumptions about the linear radiometric response function, static scenes, and no saturation. We confirmed that the superposition principle holds true for our setup, i.e., when observing objects in daily life with a normal color camera, but it does not necessarily hold because fluorophores saturate or bleach, especially in fluorescence imaging for cell and molecular biology.

In general, the superposition principle holds true for more than three primary light source colors. We denote the number of primary light source colors by *C*, and let us assume that the spectrum of a novel light source color is represented by a linear combination of the spectra of the *C* primary light source colors. Then, the image i of a scene under the novel light source spectrum is represented by the linear combination of the images $i_{c}\;\left(c=1,2,3,\ldots ,C\right)$ of the scene under the primary light source colors as

(2) $$i=\sum_{c=1}^{C}w_{c}i_{c},$$

where $w_{c}$ are the coefficients of the linear combination.

The above Equations (1) and (2) mean that we can synthesize photo-realistic images of a scene from novel viewpoints and under novel light source colors/spectra if the images of the scene under three/more than three primary light source colors from novel viewpoints can be recovered. Therefore, we extend the NeRF-based novel-view synthesis by incorporating the superposition principle of light in order to achieve fluorescent novel-view synthesis under novel light source colors/spectra. Note that our proposed method can accurately reproduce the colors of reflective materials as well because the superposition principle holds true for most materials.

### 3.2. Pipeline

**Figure 2:**
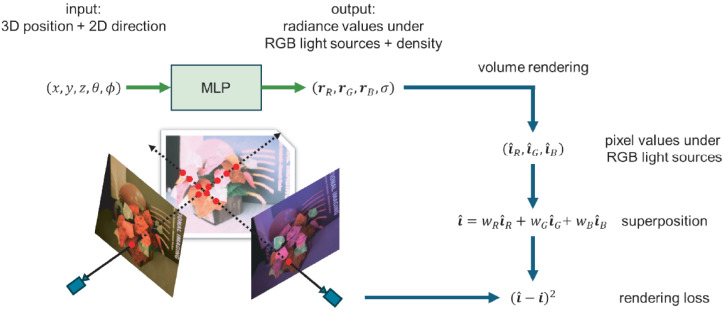
The illustration of the overall training procedure for the case with three primary light source colors (C=3). The loss is the squared error between the observed pixel values and the pixel values computed via volume rendering and the superposition principle.

The following is the pipeline of our proposed method. Figure 2 illustrates the overall training procedure for the case with three primary light source colors (C=3).

**A.**  **Data Acquisition**

**Figure 3:**
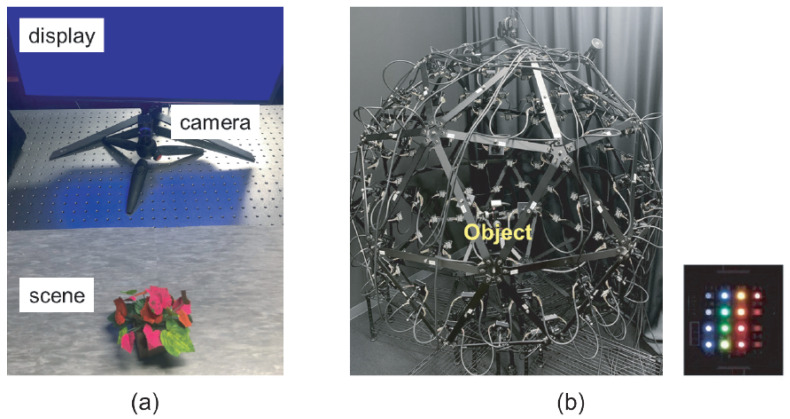
Our setups: we use (**a**) a color LCD with three primary colors and (**b**) a multi-spectral light stage with more than three primary light source colors.

First, we capture a sparse image sequence of a scene of interest under varying light source colors/spectra from varying viewpoints by moving a single color camera. For the case with three primary light source colors (C=3), we can use a color display (or a projector) as a lighting system, as shown in Figure 3a, for example. For the case with more than three light source colors (C>3), we can use an LED-based multi-spectral light stage [45,46,47,48,49] as a lighting system, as shown in Figure 3b, for example.

**B.**  **Preprocessing**

Second, as preprocessing, we estimate the camera pose and the light source color/intensities of each frame in the image sequence. In order to estimate the camera poses, we make use of structure from motion in a similar manner to the original NeRF [3].

For the case with three primary light source colors (C=3), in order to estimate the light source color, we capture three reference images of the scene under three primary light source colors (R, G, and B). Then, we manually select a patch in the scene whose colors are due to pure reflection and estimate the light source color of each frame from the average color observed over the patch via the least-squares method. Specifically, we compute the coefficients of the linear combination $w_{R}$, $w_{G}$, and $w_{B}$ by solving

(3) $$\tilde{i}=w_{R}{\tilde{i}}_{R}+w_{G}{\tilde{i}}_{G}+w_{B}{\tilde{i}}_{B}.$$

Here, $\tilde{i}$, ${\tilde{i}}_{R}$, ${\tilde{i}}_{G}$, and ${\tilde{i}}_{B}$ are the pixel values of the patch in the captured image and the reference images, respectively.

For the case with more than three light source colors (C>3), we assume that the camera and the lighting system are synchronized, and then the intensities of the primary light sources of each frame are known. This is because the estimation of the light source intensities results in an under-constrained problem when C>3.

**C.**  **Training**

Third, similar to the original NeRF [3], we represent a scene of interest by using an MLP. The input to the MLP is a 5D coordinate (x,y,z,θ,ϕ), but the output is $\left(r_{R},r_{G},r_{B},\sigma \right)$ for the case with three primary light source colors (C=3) and $\left(r_{1},r_{2},\ldots ,r_{C},\sigma \right)$ for the case with more than three primary light source colors (C>3). Here, $\left(r_{R},r_{G},r_{B}\right)$/$\left(r_{1},r_{2},\ldots ,r_{C}\right)$ are the view-dependent radiance values emitted from the point (x,y,z) to the direction (θ,ϕ) under the three/more than three light source colors.

We train the MLP by using the acquired image sequence and the estimated camera poses and light source colors/intensities. The details of the network architecture and its training are explained in Section 3.3.

**D.**  **Image Synthesis**

Finally, we synthesize the images of the scene from novel viewpoints and under novel light source colors/spectra. Specifically, we synthesize three/more than three images from a desired viewpoint under three/more than three primary light source colors via volume rendering. Then, we obtain the image under a desired light source color/spectrum by linearly combining those three/more than three images according to the color/spectrum of the novel light source.

### 3.3. Network

**Architecture:**

**Figure 4:**
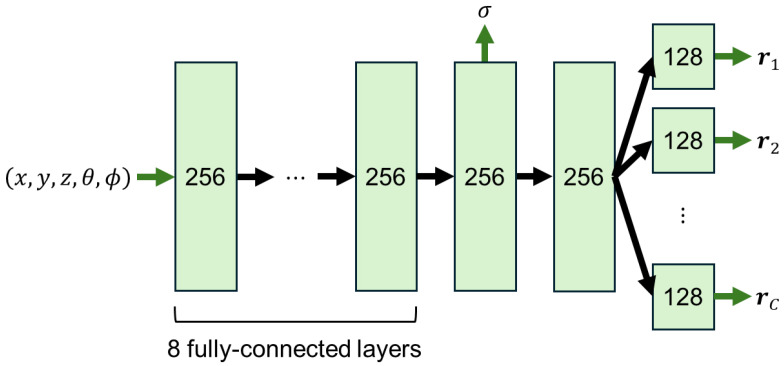
The architecture of our proposed network. Our network has *C* output layers in order to predict the radiance values under the *C* primary light source colors but shares a single volume density among the primary light source colors.

Figure 4 shows the architecture of our proposed network. Our network is similar to the original NeRF in that it has eight fully connected layers using ReLU activations and 256 channels per layer. However, in order to predict the radiance values under *C* (≥3) primary light source colors, our network has not a single but *C* output layers instead. Note that our network outputs a single volume density. In other words, it shares the volume density among the primary light source colors.

**Training:**

Figure 2 illustrates the training procedure for the case with three primary light source colors (C=3), for example. The loss is the total squared error between the rendered and true pixel values for both coarse and fine renderings:

(4) $$L=\sum_{r\in R}\left[{\left({\hat{i}}_{c}\left(r\right)-i\left(r\right)\right)}^{2}+{\left({\hat{i}}_{f}\left(r\right)-i\left(r\right)\right)}^{2}\right].$$

Here, *r* is a ray in the set of rays R in each batch, and i(r), ${\hat{i}}_{c}\left(r\right)$, and ${\hat{i}}_{f}\left(r\right)$ are the ground truth pixel value and the pixel values computed via volume rendering with coarse and fine samplings and the superposition principle, respectively.

## 4. Experiments with Three Primary Colors

In order to confirm the effectiveness of our proposed method with three primary light source colors, we conducted a number of experiments using real images. In this section, we report the experimental results using a color LCD, the setup of our experiments, the results of our proposed method, and the comparison with other methods.

### 4.1. Setup

As shown in Figure 3a, we illuminated scenes of interest by using a color LCD and then captured the image sequences of those scenes under varying colors displayed on the LCD. In our experiments, three colors (C, M, and Y) were displayed in turn (we experimentally confirmed that CMY slightly works better than RGB in a similar manner to illumination multiplexing [44,50]).We used an LCD of 439P9 from Phillips and a color camera of BFS-U3-27S5C-C from FLIR. We used COLMAP [51,52] for estimating the camera poses. We confirmed that the radiometric response function of the camera is linear.

**Figure 5:**
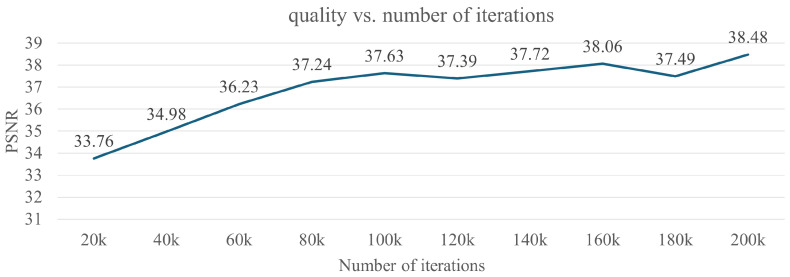
Results of quality vs. number of iterations.

In our experiments, we set the batch size to 2048 rays, and each ray was sampled at $N_{c}=64$ coordinates in the coarse volume and $N_{f}=128$ additional coordinates in the fine volume. We used the Adam optimizer [53] with a learning rate that begins at $5\times 10^{-3}$ and decays exponentially to $5\times 10^{-5}$. We trained on a PC using an NVIDIA RTX 3090 GPU, an Intel Core i9-1085k CPU, and 64 GB of RAM, with 80k epochs per scene. We evaluated the generation quality of our method using PSNR over a range of iteration numbers from 20k to 200k. Figure 5 shows the results obtained with our method. As we can see from Figure 5, the quality generated by our method becomes stable after more than 80k iterations. Therefore, all the results in our paper are based on the 80k iterations.

### 4.2. Results

We captured four image sequences by *continuously* moving a camera around four scenes with fluorescent objects. For qualitative evaluation, we used a part of the captured images (about 200 images) for training each scene and then synthesized the rest of them. Note that we have a single image under a single light source, with a single color per viewpoint, since all the images have different viewpoints.

**Figure 6:**
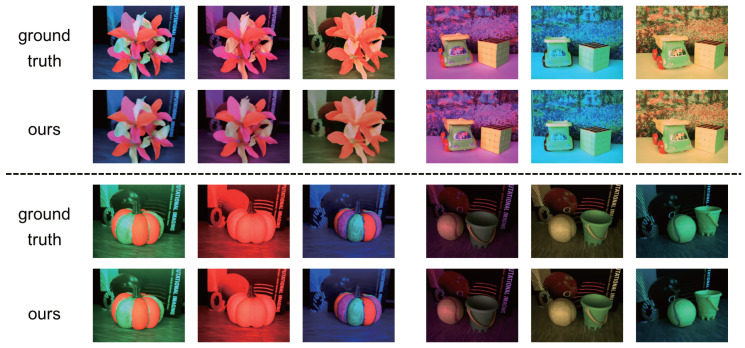
The results using the image sequences captured with an LCD and a continuously moving camera: the ground truth images and the images synthesized by using our proposed method. The generation results of four scenes at different viewpoints under novel light source colors are presented respectively.

Figure 6 shows the images from novel viewpoints and under novel light source colors: the ground truth images and the images synthesized by using our proposed method. We can find various fluorescent objects in those scenes since the colors of those objects are different from the light source colors, which are similar to the color of the background, due to the Stokes shift. We can see that our method can accurately reproduce the colors of fluorescent objects, in particular when light source colors have short-wavelength components (blue and green).

### 4.3. Comparison

We compared the performance of our proposed method with that of the following three methods:

- **W-NeRF**: the combination of the original NeRF [3] and the white balance adjustment. Specifically, an image from a novel viewpoint under white light source is synthesized by using the original NeRF, and then the color of the image is changed according to a novel light source color via the white balance adjustment.
- **RGB-NeRF**: three NeRFs, each of which is trained by using the images taken from varying viewpoints but under a fixed light source color (R, G, or B). Specifically, the three images from a novel viewpoint and under the three light source colors are separately synthesized by using the three NeRFs, and then the image under a novel light source color is synthesized by linearly combining the three images.
- **NeRD [16]**: one of the state-of-the-art techniques for scene recovery, i.e., for decomposing a scene into its shape, reflectance (BRDF), and illumination. We can synthesize the images of the scene from novel viewpoints under novel lighting environments by using those properties of the scene.

The computational times of Ours, W-NeRF, RGB-NeRF, and NeRD for pure training are about 5, 4, 12, and 6 h, respectively. The RGB-NeRF is slower than the others simply because it trains three MLPs. On the other hand, the computational times of Ours, W-NeRF, RGB-NeRF, and NeRD for volume rendering from a novel viewpoint are about 34, 21, 64, and 16 s, respectively. This is because W-NeRF and NeRD/Ours and RGB-NeRF synthesize a single image under a white light source/three images under the three primary light source colors.

**Figure 7:**
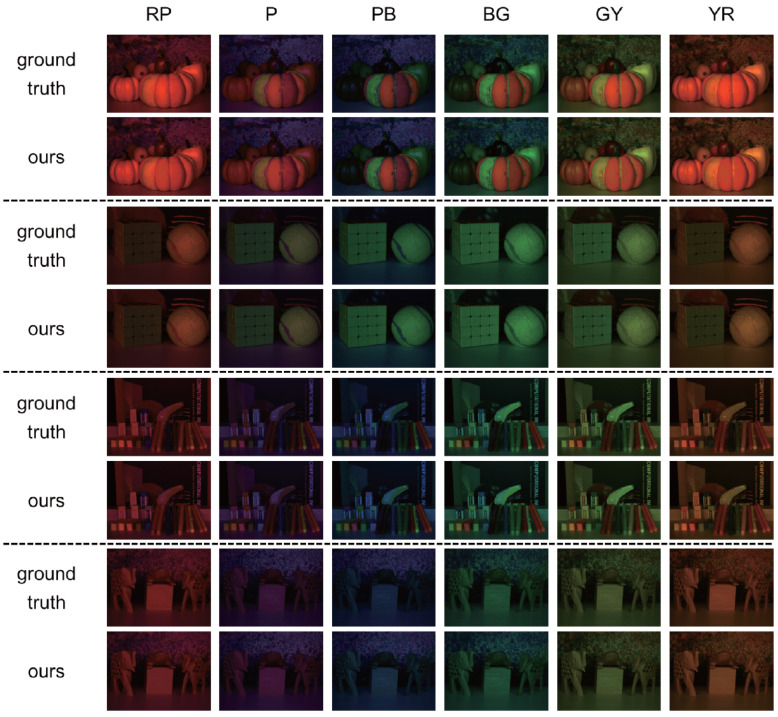
The results using the image sequences captured with an LCD and a discretely moving camera: the ground truth images and the images synthesized by using our proposed method. The generation results of four scenes at different viewpoints under the RP, P, PB, BG, GY, and YR light source colors are presented, respectively.

In contrast to Section 4.2, for the sake of fair comparison, we captured four image sequences by *discretely* moving a camera around four scenes: three scenes with fluorescent objects and one scene without them. Specifically, we captured 13 images under different light source colors per viewpoint: 10 colors (RP, P, PB, B, BG, G, GY, Y, YR, and R) from the Munsell color system and an additional three colors (C, M, and W). Figure 7 shows the results using the image sequences of those four scenes: the ground truth images and the images synthesized by using our proposed method. Those results also show the effectiveness of our method.

As summarized in Table 1, we trained all the methods by using 120 images; our proposed method, W-NeRF, RGB-NeRF, and NeRD used the images captured under the light source colors of (C, M, Y), W, (R, G, B), and W, respectively. The images captured under the rest of those colors (RP, P, PB, BG, GY, YR) from different viewpoints were used for evaluation: 90 (=15 viewpoints × 6 colors) images in total. Note that all the methods shared the same camera poses and light source colors computed as described at Preprocessing in Section 3.2.

**Our method vs. W-NeRF:**

**Figure 8:**
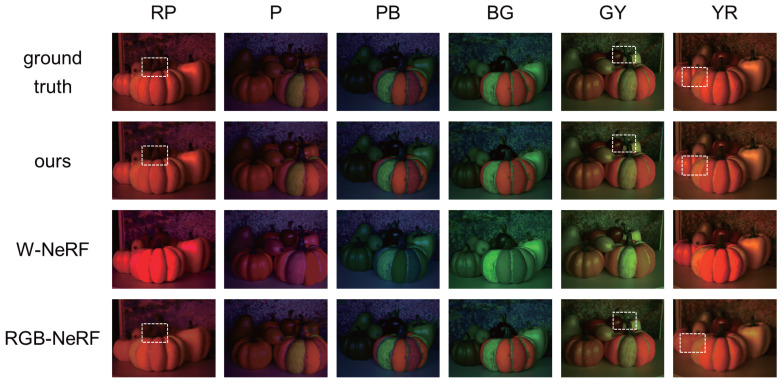
The comparison using the image sequences captured with an LCD and a discretely moving camera: the ground truth images and the images synthesized by using our proposed method, W-NeRF, and RGB-NeRF. Four different scenes were demonstrated, showing the generation results of different viewpoints under the light source colors of RP, P, PB, BG, GY, and YR. The dashed box highlights regions with noticeable texture differences.

First, Figure 8 shows the synthesized images of the first scene (painted pumpkin) from novel viewpoints and under novel light source colors. We can see that W-NeRF does not work well; the colors of fluorescent objects are almost the same as the light source colors (and the background color). On the other hand, we can see that our proposed method can accurately reproduce the colors of fluorescent objects. Table 2 shows the PSNRs and SSIMs of those methods for the four scenes. We can see that our method is quantitatively superior to W-NeRF for the three scenes with fluorescent objects. In Appendix B, we show the reason why we cannot reproduce the colors of fluorescent objects under varying light source colors via the white balance adjustment.

**Our method vs. RGB-NeRF:**

**Figure 9:**
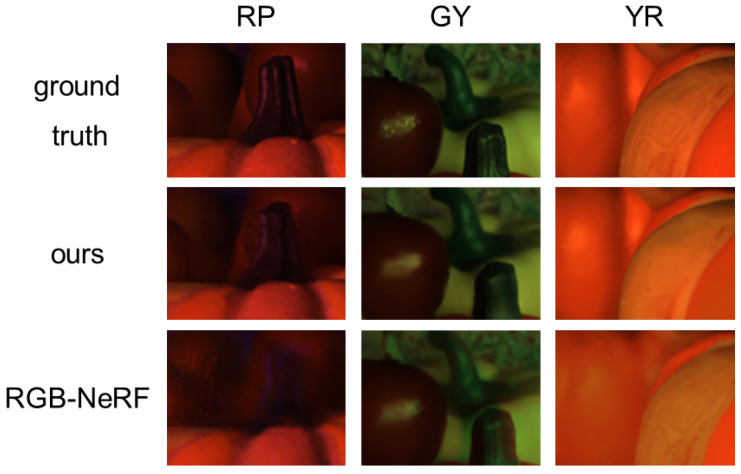
The close-up of the images in Figure 8 under the light source colors of RP, GY, and YR. It is clearly observable that our method differs from RGB-NeRF in terms of the detailed textures.

**Figure 10:**
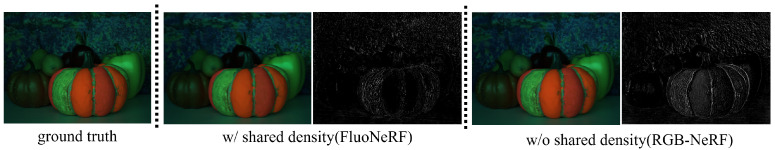
The results of our FluoNeRF with shared density and RGB-NeRF with independent density.

Second, Figure 8 shows that the colors of fluorescent objects reproduced by using RGB-NeRF are similar to those reproduced by using our proposed method. However, the close-up of those images in Figure 9 shows that RGB-NeRF causes misregistration among the images synthesized under the three light source colors: we can observe blur and pseudo colors around some edges. This is because RGB-NeRF does not share volume densities and then separately synthesizes images from novel viewpoints. Figure 10 shows the difference (×10) in images between ours/RGB-NeRF and the ground truth. The brighter pixels have larger errors. We can clearly see that RGB-NeRF has larger errors near the depth edges.

On the other hand, no misregistration is observed in the results using our method with shared volume densities. Table 2 also shows that our method performs better than RGB-NeRF for the three scenes with fluorescent objects.

**Our method vs. NeRD:**

**Figure 11:**
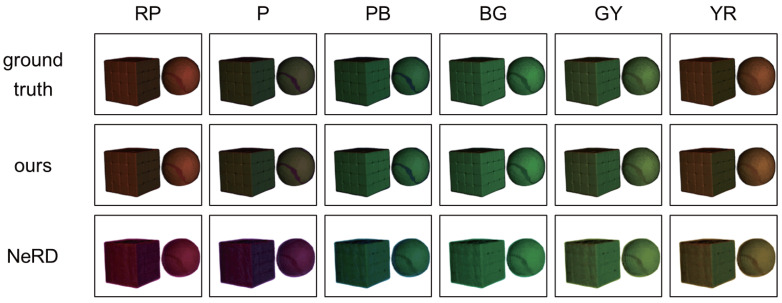
Qualitative comparison of our proposed method using three primary colors with NeRD. The generation results of different viewpoints under the light source colors, such as RP, P, PB, BG, GY, and YR, were compared.

Third, Figure 11 shows the synthesized images of the second scene (cube & ball) from novel viewpoints and under novel light source colors. Here, we show only the results of the foreground for fair comparison because NeRD requires a foreground mask for scene decomposition. We can see that NeRD does not work well for light source colors such as RP, P, and YR. This is because NeRD assumes wavelength-preserving reflective materials and cannot represent the Stokes shift due to fluorescence. Table 3 quantitatively shows that our proposed method works better than NeRD.

**Reflective objects:**

Finally, as a byproduct, we can see that our proposed method is superior to W-NeRF even for the fourth scene (woodwork) from Figure 8 and Table 2. This is because W-NeRF, i.e., the combination of the original NeRF and the white balance adjustment, assumes a narrow-band camera as explained in Appendix A, but the assumption is not necessarily true for consumer cameras.

## 5. Experiments with More than Three Primary Colors

In order to confirm the effectiveness of our proposed method, with more than three primary light source colors, we conducted a number of experiments using real images. In this section, we report the experimental results using a multi-spectral light stage: the setup of our experiments, the results of our method, and the comparison with other methods.

### 5.1. Setup

In contrast to the experiments with a color LCD in Section 4, we used our multi-spectral light stage termed Kyutech-OU Light Stage II [48], shown in Figure 3b, as a lighting system with more than three primary colors. The light stage consists of 128 LED clusters at different positions, and each cluster has 16 narrow-band LEDs with different peak wavelengths. We captured the image sequence of a scene of interest by discretely moving the same color camera used in Section 4 under varying light source spectra. Specifically, we captured a set of images from a single camera position by turning on all the LEDs with a single peak wavelength at the upper hemisphere of the light stage in order.

We tested four scenes with fluorescent objects and five novel light sources, i.e., 20 combinations in total. The light sources used in our experiments were a white LED, a normal incandescent bulb, a red incandescent bulb, a green incandescent bulb, and a blue incandescent bulb.

### 5.2. Results

We trained our proposed method by using 150 images, as summarized in Table 4. Since light with shorter/longer wavelengths than visible light is more/less important for reproducing the colors of fluorescent objects due to the Stokes shift, we selected 12 narrow-band LEDs from 16. Specifically, we used 13 images under #1 to #6 and 12 images under #7 to #12, where #1 to #12 stand for the LEDs with the shortest (near UV) to the longest (red) peak wavelengths.

**Figure 12:**
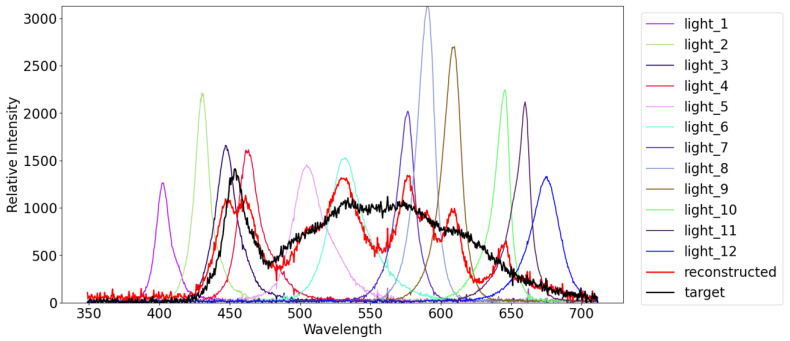
The spectral intensities of the 12 LEDs of the light stage and the measured/reconstructed spectral intensities of the white LED. Among them, the curves of numbers #1 to #12 represent the 12 LEDs of the light stage, the black curve represents the measured spectral intensities of the white LED, and the red curve represents the reconstructed spectral intensities of the white LED.

As discussed in Section 3.1, in order to synthesize the image of a scene under a novel light source on the basis of the superposition principle, we need to compute the coefficients of the linear combination $w_{c}\;\left(c=1,2,3,\ldots ,C\right)$ in Equation (2). In our experiments, we measured the spectral intensities of the LEDs of the light stage and the novel light sources by using a spectrometer BRC115P-V-ST1 from B&W Tek. Then, we assume that the spectral intensity of a novel light source l is approximately represented by the linear combination of the spectral intensities of the LEDs $l_{c}$ and solve

(5) $$l=\sum_{c=1}^{C}w_{c}l_{c}$$

with respect to $w_{c}$ via the least squares method. In Figure 12, the thin lines stand for the spectral intensities of the 12 LEDs of the light stage, and the thick black and red lines stand for the measured and reconstructed spectral intensities of the white LED, respectively. We can see that the spectral intensity of the novel light source is approximately represented by using the 12 LEDs.

**Figure 13:**
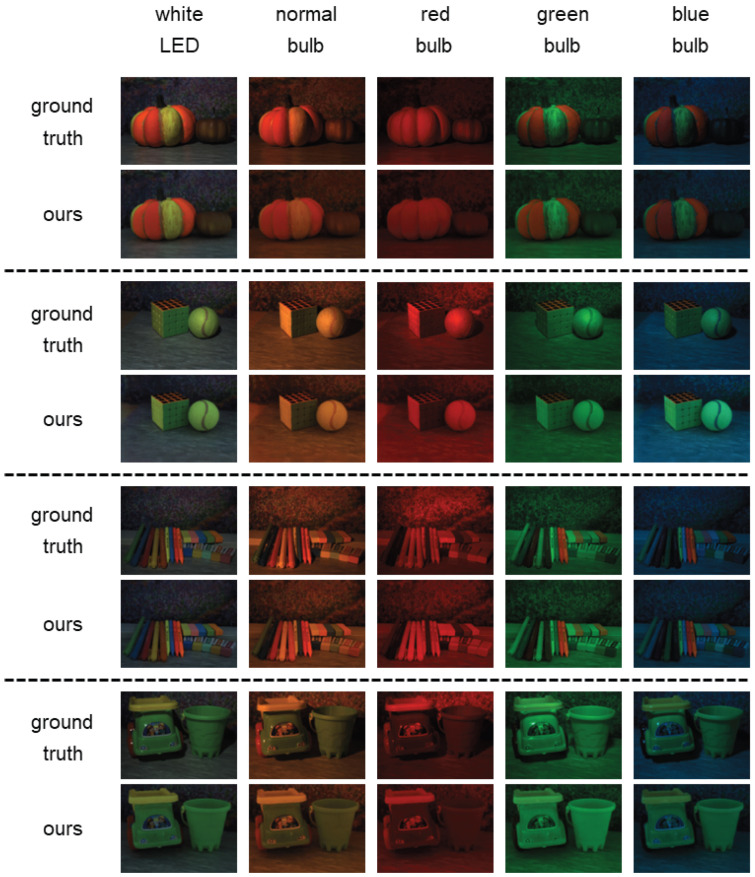
The results using the image sequences captured with a multi-spectral light stage: the ground truth images and the images synthesized by using our proposed method. Four different scenes were demonstrated, showing the generation results of different viewpoints under the light sources of white LED, nomal bulb, red bulb, green bulb, blue bulb. Additional results are provided in the Supplementary Material Video S1.

Figure 13 shows the images of the four scenes seen from novel viewpoints and under the five novel light sources. We can find various fluorescent objects in those scenes since the colors of those objects are different from the light source colors due to the Stokes shift. We can see qualitatively that our proposed method can accurately reproduce the colors of fluorescent objects, in particular when the light source colors are blue and green. Note that the shadings are different in the ground truth images and the images synthesized by using our method. This is because the spatial distributions of light sources are different: a single novel light source vs. the LEDs located at the upper hemisphere of the light stage.

### 5.3. Comparison

We compared the performance of our proposed method with that of W-NeRF and RGB-NeRF, as explained in Section 4.3. The number of training images for those methods is the same as that for our method. We captured 150 images by turning #3, #6, and #12 on at the same time for W-NeRF, and 50 images by turning one of #3, #6, and #12 on for RGB-NeRF, as summarized in Table 4.

As discussed in the previous section, the shadings are different in the ground truth images and the images synthesized from the images captured with the light stage by using our proposed method, W-NeRF, and RGB-NeRF.

**Figure 14:**
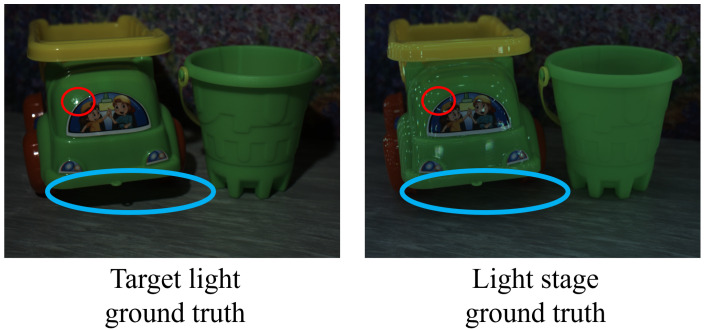
Comparison between the image captured under the target light and the synthesized light stage ground truth. The synthesized ground truth is reconstructed by a weighted combination of 12 LED-based images as described in Equation (5). Red circles indicate highlight regions, while blue circles indicate shadow regions.

**Figure 15:**
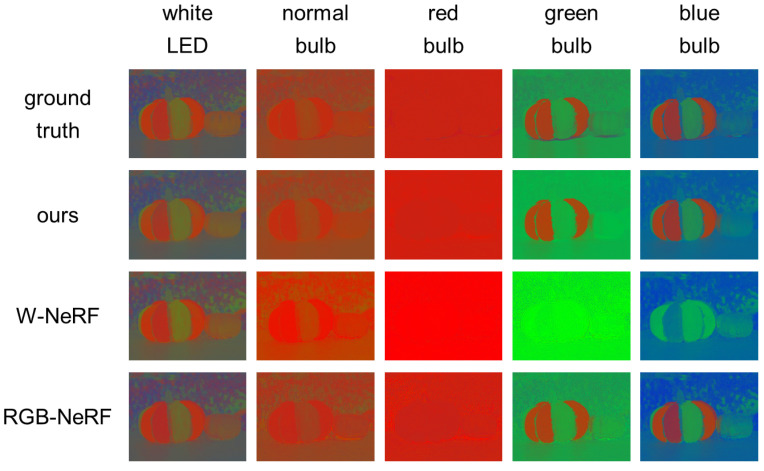
The comparison using the image sequences captured with a multi-spectral light stage: the ground truth images and the images synthesized by using our proposed method, W-NeRF, and RGB-NeRF. The RGB pixel values are converted to normalized colors according to Equation (6).

Due to the different spatial distributions of the target light source and the light stage, the captured images exhibit different shadows, shading, and highlights (indicated by blue and red circles in Figure 14). While these lead to visual discrepancies, the spectral accuracy is independently validated by the chromaticity comparison in Figure 15.

Since the point of our method is to accurately reconstruct the colors of fluorescent objects under varying light source spectra, we compare the colors of the ground truth and synthesized images. Specifically, we compute the normalized color $i^{\prime }$ at each pixel as

(6) $$i^{\prime }=\frac{255}{i_{R}+i_{G}+i_{B}}\left(\begin{array}{c} i_{R}\\ i_{G}\\ i_{B} \end{array}\right)$$

from the pixel value ${\left(i_{R},i_{G},i_{B}\right)}^{⊤}$ for an 8-bit image, and then compare the normalized colors in the ground truth and synthesized images.

**Our method vs. W-NeRF:**

First, we compare the performance of our proposed method with W-NeRF. Figure 15 shows the normalized colors of the first scene (painted pumpkin) seen from a novel viewpoint and under novel light sources. We can see that W-NeRF does not work well; the colors of fluorescent objects are almost the same as the light source colors, i.e., the colors of background reflective objects. On the other hand, we can see that our method, based on the superposition principle of light, can accurately reproduce the colors of fluorescent objects. The difference between ours and W-NeRF is remarkable under the green and blue incandescent bulbs. The PSNRs and SSIMs in Table 5 quantitatively show that our method is superior to W-NeRF.

**Our method vs. RGB-NeRF:**

Second, we compare the performance of our proposed method with that of RGB-NeRF. In Figure 15, we can see that RGB-NeRF using three primary colors can reproduce the colors of fluorescent objects better than W-NeRF. However, we can see that our method using more than three (12) primary colors performs better than RGB-NeRF. The PSNRs and SSIMs in Table 5 quantitatively show that our method outperforms W-NeRF. Those results show the effectiveness of using more than three primary colors for reproducing the colors of fluorescent objects under common light sources.

## 6. Conclusions and Future Work

In this paper, we propose a method for synthesizing photo-realistic images of scenes with fluorescent objects from novel viewpoints and under novel lighting colors and spectra. In order to handle the Stokes shift of fluorescence, we extended the novel-view synthesis based on NeRF by incorporating the superposition principle of light; our proposed method captures a sparse set of images of a scene from varying viewpoints and under varying lighting colors or spectra with active light sources such as a color display or a multi-spectral light stage and then synthesizes photo-realistic images of the scene without explicitly modeling its geometric and photometric models. We conducted a number of experiments using real images and confirmed that our method works better than the existing methods. Moreover, we showed that the extension of our method using more than three primary colors enables us to reproduce the colors of fluorescent objects under common light sources.

The extension of our proposed method for arbitrary light source directions is one of the directions of our future work. Since NeRF-based methods require a significant amount of computational resources, efficient representation and rendering based on 3D Gaussian splatting [33] is another direction of our future work. This study focuses on static scenes and opaque materials as an initial step toward establishing a fundamental framework. Therefore, complex scenarios such as biological samples or dynamic objects are beyond the current scope.

## Figures and Tables

**Table 1:** The number of images used for training each method: our proposed method with three primary colors, W-NeRF, RGB-NeRF, and NeRD. We used 120 images captured under the light source colors of (C, M, Y), W, (R, G, B), and W, respectively.

Method	Number of Images
Ours	40 (C) + 40 (M) + 40 (Y)
W-NeRF	120 (W)
RGB-NeRF	40 (R) + 40 (G) + 40 (B)
NeRD	120 (W)

**Table 2:** Quantitative comparison of our proposed method using three primary colors with W-NeRF and RGB-NeRF for three scenes with fluorescent objects and one scene without fluorescence.

Scene	Method	PSNR ↑	SSIM ↑
	Ours	**35.36**	**0.914**
Painted Pumpkin	W-NeRF	29.86	0.606
	RGB-NeRF	34.28	0.878
	Ours	**41.10**	**0.967**
Cube & Ball	W-NeRF	29.82	0.670
	RGB-NeRF	37.48	0.960
	Ours	**37.67**	**0.963**
Stationery	W-NeRF	30.36	0.670
	RGB-NeRF	32.87	0.875
	Ours	**39.23**	**0.959**
Woodwork	W-NeRF	31.12	0.664
(w/o fluorescence)	RGB-NeRF	35.13	0.854

↑ indicates higher is better. Bold text indicates the best results.

**Table 3:** Quantitative comparison of our proposed method using three primary colors with NeRD for the second scene.

Scene	Method	PSNR ↑	SSIM ↑
Cube & Ball	Ours	**33.04**	**0.984**
(foreground only)	NeRD	24.06	0.813

↑ indicates higher is better. Bold text indicates the best results.

**Table 4:** The number of images used for training each method: our proposed method, with more than three primary colors, W-NeRF, and RGB-NeRF. We used the LEDs of our multi-spectral light stage: #1 (near UV) to #12 (red).

Method	Number of Images
	13 (#1) + 13 (#2) + 13 (#3) + 13 (#4)
Ours	+ 13 (#5) + 13 (#6) + 12 (#7) + 12 (#8)
	+ 12 (#9) + 12 (#10) + 12 (#11) + 12 (#12)
W-NeRF	150 (#3 + #6 + #12)
RGB-NeRF	50 (#3) + 50 (#6) + 50 (#12)

**Table 5:** Quantitative comparison of our proposed method using more than three primary colors with W-NeRF and RGB-NeRF for four scenes with fluorescent objects.

Scene	Method	PSNR ↑	SSIM ↑
	Ours	**30.59**	**0.894**
Painted Pumpkin	W-NeRF	17.69	0.495
	RGB-NeRF	28.52	0.759
	Ours	**31.24**	**0.904**
Cube & Ball	W-NeRF	16.41	0.501
	RGB-NeRF	26.23	0.728
	Ours	**30.67**	**0.869**
Stationery	W-NeRF	16.53	0.446
	RGB-NeRF	28.52	0.759
	Ours	**30.88**	**0.905**
Truck & Bucket	W-NeRF	17.86	0.501
	RGB-NeRF	27.31	0.728

↑ indicates higher is better. Bold text indicates the best results.

## Data Availability

The raw data supporting the conclusions of this article will be made available by the authors on request.

## Supplementary Materials

The following supporting information can be downloaded at: https://www.mdpi.com/article/10.3390/jimaging12010016/s1, Video S1: Pipeline Overview and Additional Results.

## Appendix A. Colors of Reflective Components

In this appendix, we show the reason why we can approximately reproduce the colors of reflective components under arbitrary light source colors via white balance adjustment. Here, we consider diffuse reflection components for the sake of simplicity, but we can derive similar conclusions for specular reflection components.

We denote the wavelength, the spectral intensity of a light source, the spectral reflectance of a surface point, and the spectral sensitivity of the *c* channel of a camera by λ, l(λ), ρ(λ), and $s_{c}\left(\lambda \right)$, respectively. Then, the pixel color d at the surface point is given by

(A1) $$d=g_{d}\left(\begin{array}{c} \int l\left(\lambda \right)\rho \left(\lambda \right)s_{R}\left(\lambda \right)d\lambda \\ \int l\left(\lambda \right)\rho \left(\lambda \right)s_{G}\left(\lambda \right)d\lambda \\ \int l\left(\lambda \right)\rho \left(\lambda \right)s_{B}\left(\lambda \right)d\lambda \end{array}\right),$$

where $g_{d}$ is the geometric term depending on the lighting and viewing directions.

When we assume a narrow-band camera, i.e., the spectral sensitivity is sharp enough around a peak wavelength, we can approximately represent the spectral sensitivity as $s_{c}\left(\lambda \right)=\delta \left(\lambda -\lambda_{c}\right)$. Here, δ() and $\lambda_{c}$ are the Dirac delta function and the peak wavelength of the *c* channel. Substituting the delta function into Equation (A1), we obtain

(A2) $$d≃g_{d}\left(\begin{array}{c} l\left(\lambda_{R}\right)\rho \left(\lambda_{R}\right)\\ l\left(\lambda_{G}\right)\rho \left(\lambda_{G}\right)\\ l\left(\lambda_{B}\right)\rho \left(\lambda_{B}\right) \end{array}\right).$$

Moreover, we assume that the light source color is white, i.e., l(λ)=1, and then we obtain

(A3) $$d≃g_{d}\left(\begin{array}{c} \rho (\lambda_{R})\\ \rho (\lambda_{G})\\ \rho (\lambda_{B}) \end{array}\right).$$

Thus, we can find that the color of the reflective components in Equation (A2) is given by element-wise multiplying the light source color ${\left(l\left(\lambda_{R}\right),l\left(\lambda_{G}\right),l\left(\lambda_{B}\right)\right)}^{⊤}$ with the reflective components under a white light source in Equation (A3). This is the reason why we can approximately reproduce the colors of reflective components under arbitrary light source colors via white balance adjustment. Note that the colors synthesized via white balance adjustment are not necessarily accurate since a narrow-band camera is assumed in Equations (A2) and (A3).

## Appendix B. Colors of Fluorescent Components

In this appendix, we explore the fluorescent model under the assumption of a narrow-band camera. Then, we show the reason why we cannot reproduce the colors of fluorescent components under arbitrary light source colors via white balance adjustment, in contrast to reflective components.

We denote the wavelength of incident light, the absorption spectrum, the wavelength of outgoing light, and the emission spectrum by λ, a(λ), $\lambda^{\prime }$, and $e(\lambda^{\prime })$, respectively. Then, the pixel color f at the point on a fluorescent surface is given by

(A4) $$f=g_{f}\int l\left(\lambda \right)a\left(\lambda \right)d\lambda \left(\begin{array}{c} \int e\left(\lambda^{\prime }\right)s_{R}\left(\lambda^{\prime }\right)d\lambda^{\prime }\\ \int e\left(\lambda^{\prime }\right)s_{G}\left(\lambda^{\prime }\right)d\lambda^{\prime }\\ \int e\left(\lambda^{\prime }\right)s_{B}\left(\lambda^{\prime }\right)d\lambda^{\prime } \end{array}\right),$$

where $g_{f}$ is the geometric term depending on the lighting and viewing directions.

In the same way as the reflective components, we assume a narrow-band camera and represent the spectral sensitivity by using the Dirac delta function. Substituting the delta function into Equation (A4), we obtain

(A5) $$f≃g_{f}\int l\left(\lambda \right)a\left(\lambda \right)d\lambda \left(\begin{array}{c} e(\lambda_{R})\\ e(\lambda_{G})\\ e(\lambda_{B}) \end{array}\right)$$

Moreover, we assume that the light source color is white, and then we obtain

(A6) $$f≃g_{f}\int a\left(\lambda \right)d\lambda \left(\begin{array}{c} e(\lambda_{R})\\ e(\lambda_{G})\\ e(\lambda_{B}) \end{array}\right).$$

Thus, we can find that the color of the fluorescent components in Equation (A4) is independent of the spectral intensity of the light source; the spectral intensity determines not the color but the intensity of a fluorescent component. In contrast to reflective components, the color of a fluorescent surface under arbitrary lighting colors in Equation (A5) is the same as that under a white light source in Equation (A6). This is the reason why we cannot reproduce the colors of fluorescent components under arbitrary light source colors via white balance adjustment.

## References

[1] Barnard K. (1999). Color constancy with fluorescent surfaces. Color Imaging Conf..

[2] Zhang C., Sato I. Separating reflective and fluorescent components of an image. Proceedings of the CVPR 2011.

[3] Mildenhall B., Srinivasan P., Tancik M., Barron J., Ramamoorthi R., Ng R. Nerf: Representing scenes as neural radiance fields for view synthesis. Proceedings of the European Conference on Computer Vision.

[4] Cai H., Feng W., Feng X., Wang Y., Zhang J. (2022). Neural surface reconstruction of dynamic scenes with monocular rgb-d camera. Neural Inf. Process. Syst..

[5] Ost J., Mannan F., Thuerey N., Knodt J., Heide F. Neural scene graphs for dynamic scenes. Proceedings of the 2021 IEEE/CVF Conference on Computer Vision and Pattern Recognition (CVPR).

[6] Park J., Florence P., Straub J., Newcombe R., Lovegrove S. Deepsdf: Learning continuous signed distance functions for shape representation. Proceedings of the 2019 IEEE/CVF Conference on Computer Vision and Pattern Recognition (CVPR).

[7] Park K., Sinha U., Barron J., Bouaziz S., Goldman D., Seitz S., Martin-Brualla R. Nerfies: Deformable neural radiance fields. Proceedings of the IEEE/CVF International Conference on Computer Vision (ICCV2021).

[8] Pumarola A., Corona E., Pons-Moll G., Moreno-Noguer F. D-nerf: Neural radiance fields for dynamic scenes. Proceedings of the 2021 IEEE/CVF Conference on Computer Vision and Pattern Recognition (CVPR).

[9] Rudnev V., Elgharib M., Smith W., Liu L., Golyanik V., Theobalt C. Nerf for outdoor scene relighting. Proceedings of the European Conference on Computer Vision.

[10] Yu H., Guibas L., Wu J. Unsupervised discovery of object radiance fields. Proceedings of the ICLR 2022.

[11] Duisterhof B., Mao Y., Teng S., Ichnowski J. Residual-nerf: Learning residual nerfs for transparent object manipulation. InProceedings of the 2024 IEEE International Conference on Robotics and Automation (ICRA).

[12] Ichnowski J., Avigal Y., Kerr J., Goldberg K. (2020). Dex-NeRF: Using a neural radiance field to grasp transparent objects. arXiv.

[13] Levy D., Peleg A., Pearl N., Rosenbaum D., Akkaynak D., Korman S., Treibitz T. Seathru-nerf: Neural radiance fields in scattering media. Proceedings of the 2023 IEEE/CVF Conference on Computer Vision and Pattern Recognition (CVPR).

[14] Sethuraman A., Ramanagopal M., Skinner K. Waternerf: Neural radiance fields for underwater scenes. Proceedings of the OCEANS 2023—MTS/IEEE U.S. Gulf Coast.

[15] Wang Z., Yang W., Cao J., Hu Q., Xu L., Yu J., Yu J. Neref: Neural refractive field for fluid surface reconstruction and rendering. Proceedings of the 2023 IEEE International Conference on Computational Photography (ICCP).

[16] Boss M., Braun R., Jampani V., Barron J., Liu C., Lensch H. NeRD: Neural reflectance decomposition from image collections. Proceedings of the IEEE/CVF International Conference on Computer Vision (ICCV2021).

[17] Boss M., Jampani V., Braun R., Liu C., Barron J., Lensch H. Neural-PIL: Neural pre-integrated lighting for reflectance decomposition. Proceedings of the 35th International Conference on Neural Information Processing Systems (NeurIPS2021).

[18] Guo Y., Kang D., Bao L., He Y., Zhang S. Nerfren: Neural radiance fields with reflections. Proceedings of the 2022 IEEE/CVF Conference on Computer Vision and Pattern Recognition (CVPR).

[19] Hasselgren J., Hofmann N., Munkberg J. (2022). Shape, light, and material decomposition from images using monte carlo rendering and denoising. Neural Inf. Process. Syst..

[20] Liu Y., Wang P., Lin C., Long X., Wang J., Liu L., Komura T., Wang W. (2023). Nero: Neural geometry and brdf reconstruction of reflective objects from multiview images. ACM Trans. Graph. (TOG).

[21] Martin-Brualla R., Radwan N., Sajjadi M., Barron J., Dosovitskiy A., Duckworth D. Nerf in the wild: Neural radiance fields for unconstrained photo collections. Proceedings of the 2021 IEEE/CVF Conference on Computer Vision and Pattern Recognition (CVPR).

[22] Wang P., Liu L., Liu Y., Theobalt C., Komura T., Wang W. Neus: Learning neural implicit surfaces by volume rendering for multi-view reconstruction. Proceedings of the 35th International Conference on Neural Information Processing Systems.

[23] Xu Y., Zoss G., Chandran P., Gross M., Bradley D., Gotardo P. Renerf: Relightable neural radiance fields with nearfield lighting. Proceedings of the 2023 IEEE/CVF International Conference on Computer Vision (ICCV).

[24] Yao Y., Zhang J., Liu J., Qu Y., Fang T., McKinnon D., Tsin Y., Quan L. Neilf: Neural incident light field for physically-based material estimation. Proceedings of the European Conference on Computer Vision.

[25] Zhang K., Luan F., Wang Q., Bala K., Snavely N. Physg: Inverse rendering with spherical gaussians for physics-based material editing and relighting. Proceedings of the 2021 IEEE/CVF Conference on Computer Vision and Pattern Recognition (CVPR).

[26] Zhang X., Fanello S., Tsai Y., Sun T., Xue T., Pandey R., Orts-Escolano S., Davidson P., Rhemann C., Debevec P. (2021). Neural light transport for relighting and view synthesis. ACM Trans. Graph. (TOG).

[27] Zhang X., Srinivasan P., Deng B., Debevec P., Freeman W., Barron J. (2021). Nerfactor: Neural factorization of shape and reflectance under an unknown illumination. ACM Trans. Graph. (TOG).

[28] Zhang Y., Sun J., He X., Fu H., Jia R., Zhou X. Modeling indirect illumination for inverse rendering. Proceedings of the 2022 IEEE/CVF Conference on Computer Vision and Pattern Recognition (CVPR).

[29] Barron J., Mildenhall B., Verbin D., Srinivasan P., Hedman P. Zip-NeRF: Anti-aliased grid-based neural radiance fields. Proceedings of the IEEE/CVF International Conference on Computer Vision (ICCV2023).

[30] Barron J., Mildenhall B., Tancik M., Hedman P., Martin-Brualla R., Srinivasan P. Mip-NeRF: A multiscale representation for anti-aliasing neural radiance fields. Proceedings of the IEEE/CVF International Conference on Computer Vision (ICCV2021).

[31] Chen Z., Li Z., Song L., Chen L., Yu J., Yuan J., Xu Y. NeuRBF: A neural fields representation with adaptive radial basis functions. Proceedings of the IEEE/CVF International Conference on Computer Vision (ICCV2023).

[32] Garbin S., Kowalski M., Johnson M., Shotton J., Valentin J. Fastnerf: High-fidelity neural rendering at 200fps. Proceedings of the 2021 IEEE/CVF International Conference on Computer Vision (ICCV).

[33] Kerbl B., Kopanas G., Leimkuehler T., Drettakis G. (2023). 3d gaussian splatting for real-time radiance field rendering. ACM Trans. Graph..

[34] Liu L., Gu J., Lin K.Z., Chua T., Theobalt C. (2020). Neural sparse voxel fields. Neural Inf. Process. Syst..

[35] Müller T., Evans A., Schied C., Keller A. (2022). Instant neural graphics primitives with a multiresolution hash encoding. ACM Trans. Graph. (TOG).

[36] Tretschk E., Tewari A., Golyanik V., Zollhöfer M., Lassner C., Theobalt C. Non-rigid neural radiance fields: Reconstruction and novel view synthesis of a dynamic scene from monocular video. Proceedings of the 2021 IEEE/CVF International Conference on Computer Vision (ICCV).

[37] Fu Y., Lam A., Sato I., Okabe T., Sato Y. Separating reflective and fluorescent components using high frequency illumination in the spectral domain. Proceedings of the 2013 IEEE International Conference on Computer Vision.

[38] Koyamatsu K., Hidaka D., Okabe T., Lensch H.P.A. Reflective and fluorescent separation under narrow-band illumination. Proceedings of the 2019 IEEE/CVF Conference on Computer Vision and Pattern Recognition (CVPR).

[39] Fu Y., Lam A., Sato I., Okabe T., Sato Y. (2015). Reflectance and fluorescence spectral recovery via actively lit rgb images. IEEE Trans. Pattern Anal. Mach. Intell..

[40] Treibitz T., Murez Z., Mitchell B., Kriegman D. Shape from fluorescence. Proceedings of the European Conference on Computer Vision (ECCV 2012).

[41] Hullin M., Hanika J., Ajdin B., Seidel H.-P., Kautz J., Lensch H. (2010). Acquisition and analysis of bispectral bidirectional reflectance and reradiation distribution functions. ACM Trans. Graph..

[42] Lyu L., Tewari A., Leimkühler T., Habermann M., Theobalt C. Neural radiance transfer fields for relightable novel-view synthesis with global illumination. Proceedings of the European Conference on Computer Vision.

[43] Sun T., Lin K., Bi S., Xu Z., Ramamoorthi R. Nelf: Neural light-transport field for portrait view synthesis and relighting. Proceedings of the EGSR2021.

[44] Schechner Y., Nayar S., Belhumeur P. A theory of multiplexed illumination. Proceedings of the Proceedings Ninth IEEE International Conference on Computer Vision.

[45] Ajdin B., Finckh M., Fuchs C., Hanika J., Lensch H. (2012). Compressive Higher-Order Sparse and Low-Rank Acquisition with a Hyperspectral Light Stage.

[46] Debevec P. The Light Stages and Their Applications to Photoreal Digital Actors. Proceedings of the SIGGRAPH Asia2012.

[47] Gu J., Liu C. Discriminative illumination: Per-pixel classification of raw materials based on optimal projections of spectral BRDF. Proceedings of the 2012 IEEE Conference on Computer Vision and Pattern Recognition.

[48] Kurachi M., Kawahara R., Okabe T. One-Shot Polarization-Based Material Classification with Optimal Illumination. Proceedings of the 20th International Conference on Computer Vision Theory and Applications.

[49] Wang C., Okabe T. Joint optimization of coded illumination and grayscale conversion for one-shot raw material classification. Proceedings of the 28th British Machine Vision Conference (BMVC2017).

[50] Park J.-I., Lee M.-H., Grossberg M., Nayar S. Multispectral imaging using multiplexed illumination. Proceedings of the 2007 IEEE 11th International Conference on Computer Vision.

[51] Schönberger J., Frahm J. Structure-from-motion revisited. Proceedings of the 2016 IEEE Conference on Computer Vision and Pattern Recognition (CVPR).

[52] Schönberger J., Zheng E., Pollefeys M., Frahm J. Pixelwise view selection for unstructured multi-view stereo. Proceedings of the European Conference on Computer Vision.

[53] Kingma D., Ba J. Adam: A method for stochastic optimization. Proceedings of the International Conference on Learning Representations (ICLR).

